# Synthesis and Antimicrobial Evaluation of Novel Pyrazolopyrimidines Incorporated with Mono- and Diphenylsulfonyl Groups

**DOI:** 10.3390/molecules24214009

**Published:** 2019-11-05

**Authors:** Amani M. R. Alsaedi, Thoraya. A. Farghaly, Mohamed R. Shaaban

**Affiliations:** 1 Department of Chemistry, Faculty of Applied Science, Umm Al-Qura University, Makkah Almukaramah 21514, Saudi Arabia; 2 Department of Chemistry, Faculty of Science, Cairo University, Giza 12613, Egypt

**Keywords:** antimicrobial activity, pyrazolopyrimidine, aminopyrazoles, microwaves, structure-activity relationship (SAR)

## Abstract

A novel series of pyrazolo[1,5-*a*]pyrimidine ring systems containing phenylsulfonyl moiety have been synthesized via the reaction of 2-(phenylsulfonyl)-1-(4-(phenylsulfonyl) phenyl)ethan-1-one, 2-benzenesulfonyl-1-(4-benzenesulfonyl-phenyl)-3-dimethylamino-propenone and 3-(dimethylamino)-1-(4-(phenylsulfonyl)phenyl)prop-2-en-1-one each with various substituted aminoazopyrazole derivatives in one pot reaction strategy. The proposed structure as well as the mechanism of their reactions were discussed and proved with all possible spectral data. The results of antimicrobial activities of the new sulfone derivatives revealed that several derivatives showed activity exceeding the activity of reference drug. Contrary to expectations, we found that derivatives containing one sulfone group are more effective against all bacteria and fungi used than those contain two sulfone groups.

## 1. Introduction

**Figure 1:**
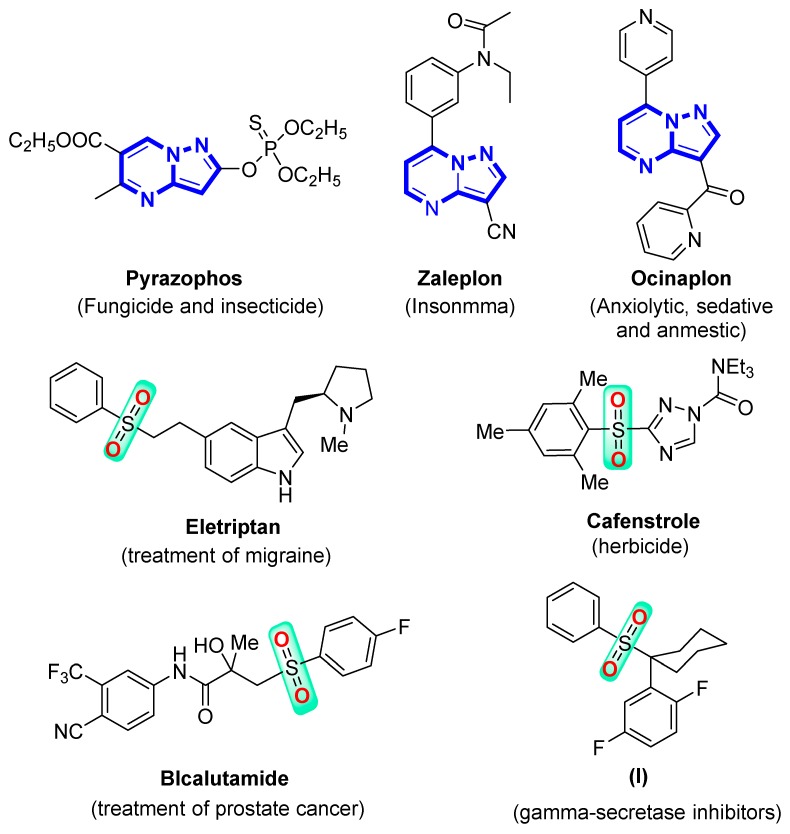
Biological activity of some pyrazolopyrimidines and drugs having benzenesulfone moiety.

Pyrazolo[1,5-*a*]pyrimidine is known to be purine analog that has protruded a vital building block for pharmaceutical drugs. It has several potent biological implementations as antischistosomal, antimetabolites in purine bio-chemical interactions, sedative and antitrypanosomal [1], AMP phosphodiesterase inhibitors [2], anxiolytic [3], benzodiazepine receptor ligands [4], KDR (kinase insert domain receptor) kinase inhibitors [5], HMG-CoA (3-hydroxy-3-methyl-glutaryl-coenzyme A reductase) reductase inhibitors [6], COX-1 (cyclooxygenase-1), COX-2 (cyclooxygenase-2) selective inhibitors [7], HCV (hepatitis C virus) inhibitors [8], serotonin 5-HT6 (5-hydroxytryptamine) receptor antagonists [9], PET (positron emission tomography) tumor imaging agents [10], kinase inhibitors [11], CCR1 (C-C chemokine receptor type 1) antagonists [12], HIV (human immunodeficiency viruses) reverse transcriptase inhibitors [13], and antifungal and antimalarial activities [14]. Many marketed drugs have pyrazolo[1,5-*a*]pyrimidine nucleus such as indiplon, zaleplon, dorsomorphin, dinaciclib, anagliptin, pyrazophos, lorediplon, and ocinaplon [15] are showed in Figure 1. Another important scaffold is benzene-sulfone moiety which present in several important pharmaceutical and agrochemical molecules due to their distinctive structural and electronic features. As for instance, molecules used as gamma-secretase inhibitors (I) [16], in migraine and prostate cancer, or as the herbicides mesotrione and cafenstrole, all feature aryl sulfone units [17] (Figure 1). Due to the specific physical and chemical properties as well as the biological activities of azobenzene dyes, they have found wide applications in the cosmetic, pharmaceutical, dyeing/textile industry, food, and analytical chemistry [18]. Many of these compounds exhibit biomedical activity because they exhibit various properties such as anti-inflammatory activity, antibacterial activity, cell protection, protease inhibitors (enzymes that play functions in many pathological disorders), or have anti-HIV activity [19,20,21]. Also, it was proved recently that azo-benzene based compounds showed a killing effect on bacteria or fungi through the interaction with their protein receptors, rather than an interaction with membrane [22,23]. On the other hand, the molecular hybridization is specialized with synthesis new compounds from combination of biologically active substances for the production of a new hybrid compound. In several cases, it generates derivatives having effective biological activities more potent than their starting moieties [24].

**Figure 2:**
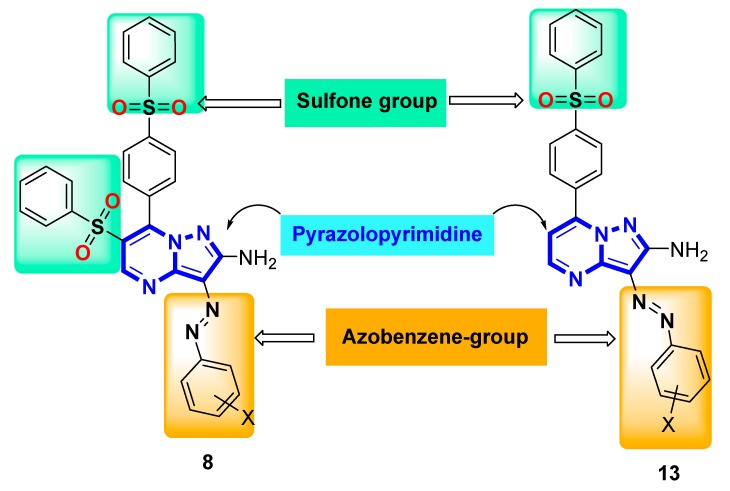
The structure of the new pyrazolopyrimidines **8** and **13**.

Inspired by these observations and in resumption of our recent research aiming at the design and synthesis of new bioactive heterocyclic systems [25,26,27,28,29,30,31], we are interested herein to design and synthesize of two new series of pyrazolo[1,5-*a*]pyrimidine derivatives, **8** and **15** (Figure 2), which have one or two arylsulfonyl and an arylazo groups to investigate their antimicrobial activities. The aim of such synthesis is to study the effect of the combination of such scaffold on the activity of these new series, as we expect to generate a potent active drug as an antimicrobial agent.

## 2. Results and Discussion

### Chemistry

**Scheme 1:**
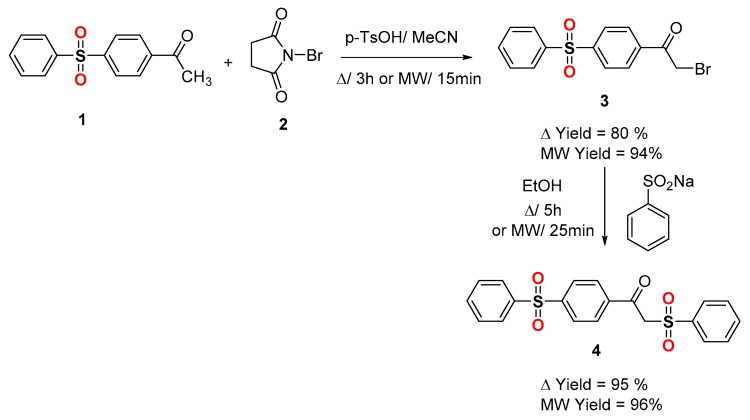
Synthesis of compound **4**.

Initially, three consecutive steps were enough to access of the hitherto unreported 3-(dimethylamino)-2-(phenylsulfonyl)-1-(4-(phenylsulfonyl)phenyl)prop-2-en-1-one **6** as a versatile multifunctional building block for construction of the targeted pyrazolo[1,5-*a*]pyrimidine derivatives. We started with modified method for α-bromination of 1-(4-(phenylsulfonyl)phenyl)ethan-1-one **1** using *N*-bromosuccinimide (NBS) in the presence of *p*-toluene sulfonic acid (*p*-TsOH) and acetonitrile as a solvent either under thermal or microwaves irradiation conditions (Scheme 1). The *α*-bromoketone **3** was obtained in excellent yield (94%) under pressurized microwave irradiation (MW) for 15 min using 400 W microwaves operating power. Then, treatment of compound **3** with sodium benzene sulfinate in ethanol under thermal as well as microwave conditions afforded the corresponding 2-(phenylsulfonyl)-1-(4-(phenylsulfonyl)phenyl)ethan-1-one **4** in high yield (Scheme 1). The suggested structure of compound **4** as illustrated in Scheme 1 was confirmed from its spectral data. The IR spectrum of compound **4** showed the carbonyl absorption signal vibrating at 1700 cm^−1^. The ^1^H NMR spectrum of compound **4** displayed the characteristic signal of the CH_2_ group which clearly appeared at *δ* 5.39 ppm in addition to the other protons that are resonating at their expected values (see experimental part). Further evidence that confirm the structure of compound **4** was supported from its ^13^C NMR which revealed fourteen carbon signals resonating at *δ* values as follows: 62.6 (CH_2_), 127.6, 127.7, 128.1, 129.2, 130.0, 130.2, 134.1, 134.3, 139.1, 139.2, 140.2, 145.3 (12 Ar-C), and 188.6 (C=O) ppm.

**Scheme 2:**
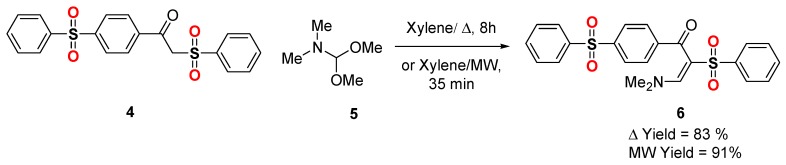
Synthesis of enaminosulfone **6**.

Thermal or microwaves heating of the 2-(phenylsulfonyl)-1-(4-(phenylsulfonyl)phenyl)ethan-1-one **4** with *N,N*-dimethylformamide-dimethylacetal (*DMF-DMA*) **5** using dry xylene as a solvent, afforded a single product identified as the corresponding 3-(dimethylamino)-2-(phenylsulfonyl)-1-(4-(phenylsulfonyl)phenyl)prop-2-en-1-one **6**, in high yields (Scheme 2). All spectral data of the formed enaminosulfone **6** were in agreement with the proposed structure. The presence of low frequency of the C=O at 1624 cm^−1^ in the IR spectrum of enaminosulfone **6** confirm its structure which attributed to the conjugation with the aromatic-C=C and the C=C of the enamine moiety. Also, the ^1^H NMR spectrum of enaminosulfone **6** clearly displayed two characteristic singlet signals for the two CH_3_ and =CH protons at 3.29 and 8.11 ppm in addition to an aromatic multiplets in the region δ 7.36–7.99 ppm. It is important to notice that the large value of chemical shift of =CH of enaminone moiety (δ = 8.11 ppm) indicated that enaminone **6** was assigned the *E*-configuration [32]. This large value of the trans-H can be attributed to the high deshielding effect of the direct interaction with SO_2_ group. While, *Z*-isomer analogous structure was reported to appear at δ 6.9 ppm [33].

**Scheme 3:**
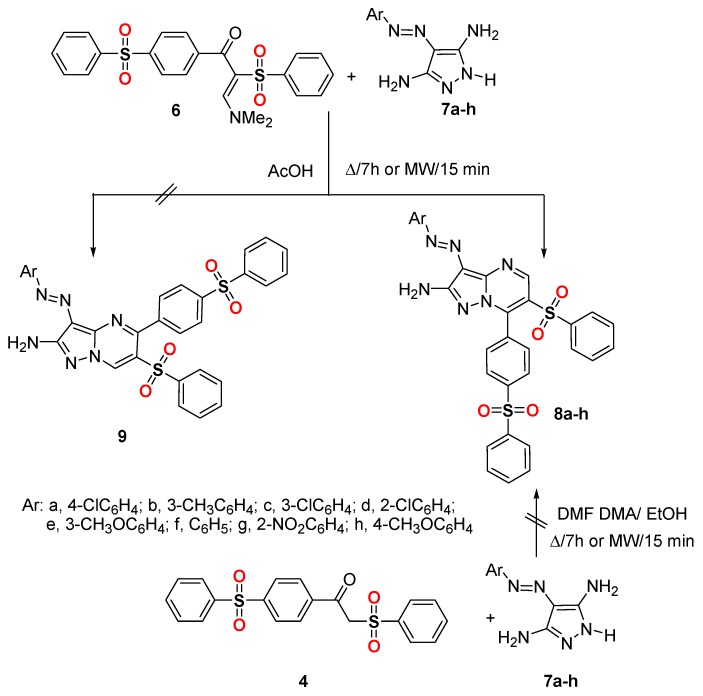
Synthesis of pyrazolopyrimidines **8a**–**h**.

The reaction of the enaminosulfone **6** with arylazodiaminopyrazole derivatives **7a**–**h** was investigated using two different pathways under thermal and microwaves irradiation conditions. Thus, when enaminosulfone **6** was treated with arylazodiaminopyrazoles **7a**–**h** in glacial acetic acid, it furnished the corresponding pyrazolopyrimidines derivatives **8a**–**h** under thermal as well as microwaves conditions (Scheme 3, Table 1).

**Figure 3:**
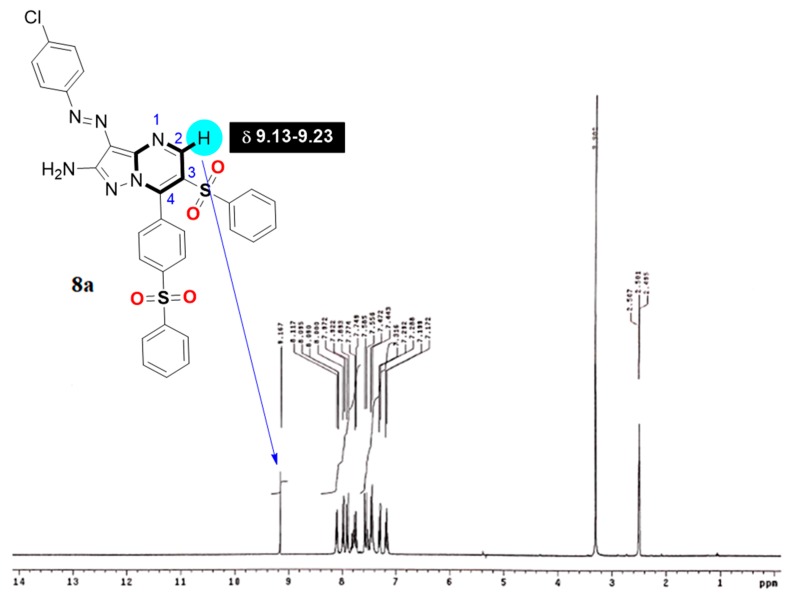
^1^H NMR of compound **8a**.

^1^H NMR of the isolated pyrazolopyrimidine derivatives gave a strong evidence for the structure **8** rather than **9**. The ^1^H NMR spectra of all derivatives **8a**–**h** were characterized with the existence of singlet signal at δ 9.13–9.23 ppm for the pyrimidine-CH-2 and not CH-4 in structure **9** as shown in Figure 3****. The presence of the pyrimidine-CH-2 at δ 9.13–9.23 ppm was confirmed previously by our group via X-ray crystallography of the same ring system [34].

**Scheme 4:**
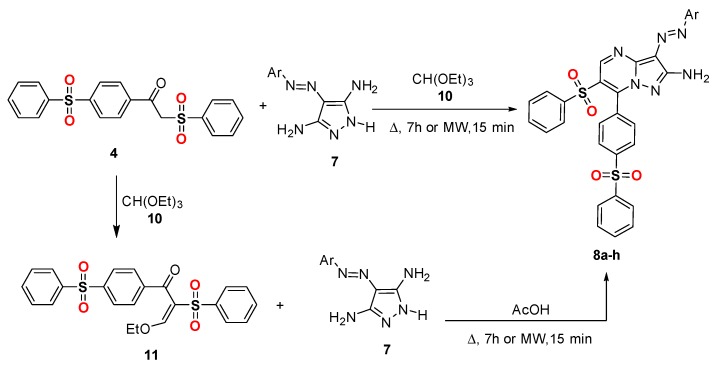
Alternative synthesis of pyrazolopyrimidines **8a**–**h**.

Also, the structure **8** was firmly established for the reaction products by an alternate synthesis. Thus, 2-(phenylsulfonyl)-1-(4-(phenylsulfonyl)phenyl)ethan-1-one **4** was condensed with triethylorthoformate **10** and subsequent condensation of the formed ethoxymethylene derivative **11** with arylazodiaminopyrazole derivatives **7a**–**h** gave products identical in all respects (m.p., mixed m.p., and spectra) with those formed from the reaction enaminosulfone **6** with pyrazoles **7**. It should be noted that, multi-components condensation of 2-(phenylsulfonyl)-1-(4-(phenylsulfonyl)-phenyl)ethan-1-one **4,** DMF-DMA, and arylazodiaminopyrazole derivatives **7a**–**h** have failed to afford the products **8a**–**h** as shown in Scheme 3. On the other hand, one pot multi-components condensation of 2-(phenylsulfonyl)-1-(4-(phenylsulfonyl)phenyl)ethan-1-one **4,** triethylortho-formate **10,** and arylazodiamino-pyrazoles **7a**–**h** afforded the products **8a**–**h** as shown in Scheme 4.

**Scheme 5:**
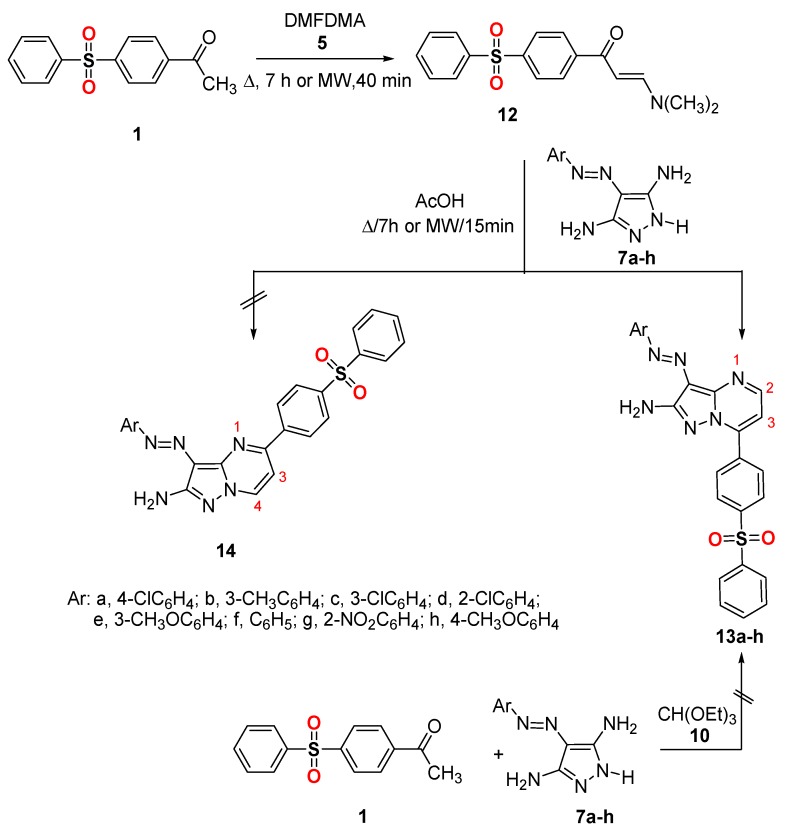
Synthesis of compounds **13a**–**h**.

In order to examine the influence of phenyl sulfonyl group pendent to pyrimidine ring on the antimicrobial activity, we have decided to synthesize a series of novel pyrazolo[1,5-*a*]pyrimidines derivatives **13a**–**h** which have an analogue structure to pyrazolo[1,5-*a*]pyrimidines derivatives **8a**–**h** by exclusion of the phenyl sulfonyl moiety in the pyrimidine ring in the pyrazolo[1,5-*a*]pyrimidine ring system. The latter can be achieved via reaction of another enaminosulfone derivative **12** with arylazodiaminopyrazoles **7a**–**h** without catalyst in glacial acetic acid under thermal as well as microwave conditions (Scheme 5). The reaction products were identified as the pyrazolo [1,5-*a*]pyrimidines derivatives **13a**–**h** and not the isomeric products **14** (Table 2).

**Figure 4:**
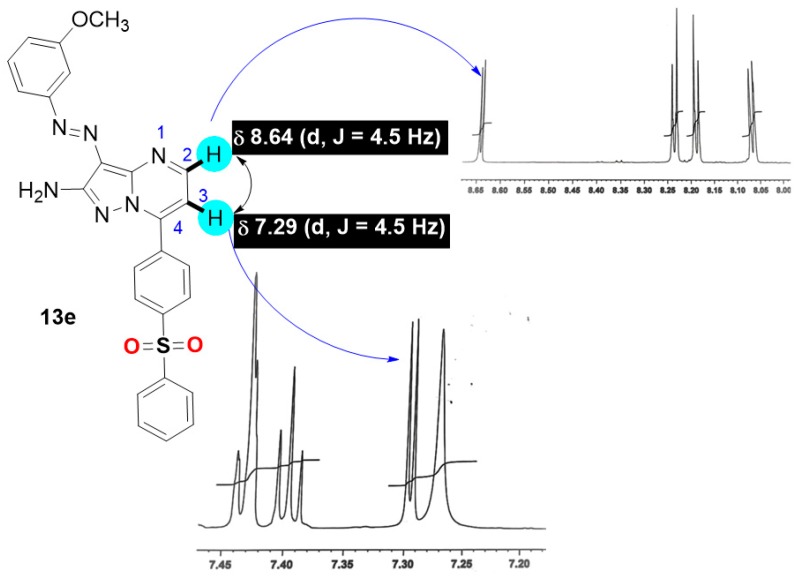
^1^H NMR of compound **13e**.

The structure of the products **13** have been confirmed based on spectral data (Figure 4). The structures of the products **13a**–**h** were established on investigation their spectral data and their elemental analyses. For example, all ^1^H NMR spectra of derivatives **13a**–**h** revealed two doublet signals (*J* ≈ 4.5 Hz) near *δ* 7.3 and 8.8 due to pyrimidine CH-3 and CH-2 protons, respectively. The other expected products **14** were ruled out on the basis of spectral data such as ^1^H NMR where the CH-4 protons was expected to resonate at low value of chemical shift *δ* in their ^1^H NMR spectrum [35] as well as the literature reports which proved the regioselectivity of such reaction by X-ray crystallographic analysis of the product [32,36].

Attempts to achieve the pyrazolo[1,5-*a*]pyrimidines derivatives **13a**–**h** via alternative pathway through multicomponent condensation of the acetyl derivative **1**, triethylorthoformate **10**, and arylazodiaminopyrazoles **7a**–**h** were not useful in this case (Scheme 5).

**Scheme 6:**
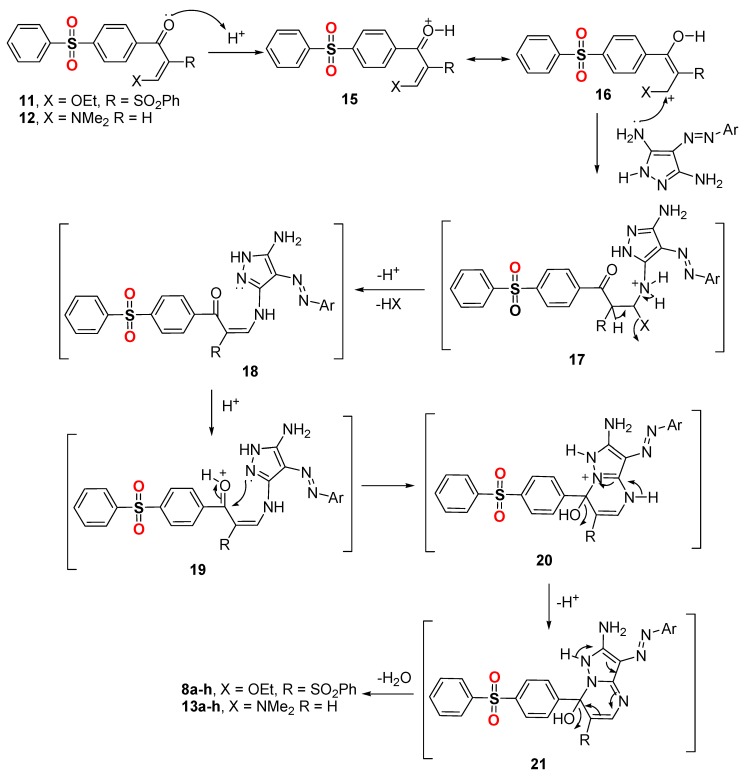
The general mechanism of formation of compounds **8a**–**h** and **13a**–**h** from ethoxymethylene derivative **11** and enaminone **12**.

From the mechanistic point of view, the multi component synthesis of **8a**–**h** was expected to proceed via Michael-type addition in acidic medium of arylazodiaminopyrazoles **7a**–**h** to the activated double bond of the ethoxymethylene derivative **11** of 2-(phenylsulfonyl)-1-(4-(phenylsulfonyl)phenyl)ethan-1-one **4** followed by loss of ethanol and subsequent intramolecular cyclization via elimination of water to afford the corresponding pyrazolo[1,5-*a*] pyrimidines derivatives **8a**–**h,** all intermediates were illustrated in Scheme 6. In the same manner and under the same acidic medium, the mechanism of the formation of pyrazolo[1,5-*a*]pyrimidines derivatives **13a**–**h** is shown in Scheme 6 which involves Michael type addition followed by cyclocondensation of the non-isolable Michael adduct **18** by loss of dimethylamine to form another three non-isolable intermediates **19**–**21** then followed by elimination of water molecule to afford the corresponding pyrazolo[1,5-*a*]pyrimidines derivatives **13a**–**h**.

## 3. Antimicrobial Activity

The antimicrobial activity of twelve new synthesized derivatives **8a**–**d**, **8h**, **13a,** and **13c**–**h** were tested against two fungi species (*Aspergillus niger* and *Geotrichum candidum*) (Table 3), four Gram-positive bacteria as well as four Gram-negative which listed in Table 4 and Table 5. The reference drugs were commonly applied antibiotics such as *Amphotericin B* (For Fungi), Ampicillin, and *Gentamicin* (For Gram-positive and Gram-negative bacteria). The first thing that can be seen from the listed results of antimicrobial activity is that all the studied derivatives did not have any effect on *P. aeruginosa* and *S. pyogenes*. For the activity of the tested derivatives against two fungi: There are three pyrazolopyrimidine sulfone derivatives **13c**, **13d**, and **13g** were found more potent than *Amphotericin* B.

In case of the activity against the Gram-positive bacteria only one derivative **13d** exceeds the activity of the reference drug *Ampicillin* against *S. epidermidis* (Table 4). While, two derivatives **13d** and **13g** revealed activity more than the reference drug used against *E. coli*. Otherwise, three pyrazolopyrimidine derivatives **13c**, **13d**, and **13g** were found more reactive than *Gentamicin* against *S. typhimurium* (Table 5).

All the other tested pyrazolopyrimidines revealed activity good to moderate against all tested microbes except *P. aeruginosa* and *S. pyogenes*.

Minimum inhibitor concentration of the three most potent pyrazolopyrimidine derivatives **13c**, **13d**, and **13g** listed in Table 6 indicated that derivative **13d** is the most effective compound.

It is clear that the presence of one sulfone group in the pyrazolopyrimidine system enhances the antimicrobial activity of the synthesized drugs, and increasing the number of sulfone groups in our case does not increase the biological activity of the compounds. Therefore, we recommend the preparation of pyrazolopyrimidine system with one sulfone group and complete the study by determining the antibacterial activity of most promising compounds on mice models 3. Materials and Methods

### 3.1. General

Melting points of synthesized compounds were measured on a Gallenkamp melting point apparatus. The infrared spectra were recorded in potassium bromide discs Shimadzu a FT-IR-4100 infrared spectrophotometer (400–4000 cm−1, JASCO, Easton, MD, USA). Nuclear magnetic resonance spectra were recorded in DMSO-*d_6_* or CDCl_3_ Using a Varian Mercury VXR-300 NMR spectrometer (JEOL, Tokyo, Japan). Chemical shifts *δ* were related to that of the used solvents. MS spectra were recorded on a Shimadzu GCMS-QP1000 EX mass spectrometer at 70 eV (Tokyo, Japan). The Microwave irradiation was carried out on a CEM mars machine (CEM Corporation, Matthews, NC, USA). CEM has several vessel types that are designed for their ovens: Closed-system vessels including the HP-500 (CEM Corporation, Matthews, NC, USA) (500 psig material design pressure and 260 °C), liners are composed of PFA, and are ideal for many types of samples. HP-500 Plus vessels are ideal for routine digestion applications. Process up to 14 high-pressure vessels per run with temperatures up to 260 °C or pressures up to 500 psi. Elemental analyses were carried out at the microanalytical center of Cairo University, Giza, Egypt.

### 3.2. Synthesis of 1-(4-benzenesulfonyl-phenyl)-2-bromo-ethanone **(3)**

#### 3.2.1. Method A: Thermal Method

To a stirred solution of 1-(4-benzenesulfonyl-phenyl)-ethanone **(1)** (2.6 g, 0.01 mol) and *p*-toluenesulfonic acid (**2**) (2 g, 0.01 mol) in acetonitrile (12 mL) was added *N*-bromosuccinimide (1.78 g, 0.01 mol) portion wise then the reaction mixture was heated for 3 h. After the reaction mixture was cooled, the solvent was evaporated and H_2_O was added and the product was extracted using chloroform to give 1-(4-benzenesulfonyl-phenyl)-2-bromo-ethanone **(3)** as pale yellow solid, yield (80%).

#### 3.2.2. Method B: Microwaves Method

In a HP-500 process vial a mixture of 1-(4-benzenesulfonyl-phenyl)-ethanone **(1)** (2.6 g, 0.01 mol) and *p*-toluenesulfonic acid (2 g, 0.01 mol) in acetonitile (12 mL) was added *N*-bromosuccinimide (1.78 g, 0.01 mol) portion wise then the vial was capped properly and was irradiated by microwaves irradiation (400 W power) using pressurized conditions at 90 °C for a period of 15 min. After the reaction mixture was cooled, the solvent was evaporated and H_2_O was added and the product was extracted using chloroform to give 1-(4-benzenesulfonyl-phenyl)-2-bromo-ethanone **(3)** as pale yellow solid, yield (94%), mp.: 115–117 °C (EtOH), IR *ύ*: 3091, 3011 (sp^2^ C-H), 2951 (sp^3^ C-H), 1705 (C=O) cm^−1^; ^1^H NMR (DMSO-*d_6_*) *δ* 4.99 (s, 2H, CH_2_), 7.64 (t, *J* = 7.65 Hz, 2H, Ar-H), 7.72 (t, *J* = 6.8 Hz, 1H, Ar-H), 8.01 (d, *J* = 7.65 Hz, 2H, Ar-H), 8.13 (d, *J* = 8.5 Hz, 2H, Ar-H), 8.18 (d, *J* = 7.65 Hz, 2H, Ar-H). ^13^C NMR (DMSO-*d_6_*) *δ*: 34.5 (CH_2_), 127.7, 127.9, 129.9, 130.0, 134.2, 137.8, 140.2, 145.1 (8 Ar-C), 191.0 (C=O). Ms *m*/*z* (%) 340 (M^+^ + 1, 12), 339 (M^+^, 20), 299 (73), 267 (68), 253 (37), 246 (48), 220 (65), 168 (32), 120 (15), 93 (100), 77 (42), and 64 (37). Anal. Calcd. For: C_14_H_11_BrO_3_S (339.20) C, 49.57; H, 3.27. Found: C, 49.46; H, 3.19%

### 3.3. Synthesis of 2-benzenesulfonyl-1-(4-benzenesulfonyl-phenyl)-ethanone **(4)**

#### 3.3.1. Method A: Thermal Method

A mixture of compound **3** (3.4 g, 0.01 mol) and sodium benzene sulfinate (1.64 g, 0.01 mol) in ethanol (12 mL) in suitable round flask was refluxed for 5 h with constant stirring. After the reaction was completed which evidenced using TLC technique, the reaction mixture was poured into ice cold water, the white precipitate was filtered off, dried, and crystallized from ethanol/*n*-hexane to give the disulfone derivative **4** as white crystals, yield (95%),

#### 3.3.2. Method B: Microwaves Method

A mixture of compound **3** (3.4 g, 0.01 mol) and sodium benzene sulfinate (1.64 g, 0.01 mol) in ethanol (12 mL) were mixed in a HP-500 process vial. The vail was capped properly and was irradiated by microwaves irradiation (800 W power) using pressurized conditions at 70 °C for a period of 25 min. The reaction mixture was the reaction mixture was poured into ice cold water, the white precipitate was filtered off, dried, and crystallized from ethanol/*n*-hexane to give the disulfone derivative **4** as white crystals, yield (96%), mp.: 180–182 °C (EtOH), IR *ύ*: 3100 (sp^2^ C-H), 2951, 2911 (sp^3^ C-H), 1700 (C=O) cm^−^^1^; ^1^H NMR (DMSO-*d_6_*) δ: 5.39 (s, 2H, CH_2_), and 7.53–8.13(m, 14H, Ar–H). ^13^C NMR (DMSO-*d_6_*) *δ*: 62.6 (CH_2_), 127.6, 127.7, 128.1, 129.2, 130.0, 130.2, 134.1, 134.3, 139.1, 139.2, 140.2, 145.3 (12 Ar-C), 188.6 (C=O). Ms *m*/*z* (%) 400 (M^+^, 48), 362 (55), 330 (100), 324 (23), 287 (63), 243 (41), 219 (41), 183 (16), 140 (40), 118 (19), 106 (9), and 41 (68). Anal. Calcd. For: C_20_H_16_O_5_S_2_ (400.47) C, 59.98; H, 4.03. Found: C, 59.84; H, 3.98%.

### 3.4. Synthesis of 2-benzenesulfonyl-1-(4-benzenesulfonyl-phenyl)-3-dimethylamino-propenone **(6)** and 1-(4-benzenesulfonyl-phenyl)-3-dimethylamino-propenone **(12)**

#### 3.4.1. Method A: Thermal Method

A mixture of 2-benzenesulfonyl-1-(4-benzenesulfonyl-phenyl)-ethanone **(4)** or 1-(4-(phenylsulfonyl)- phenyl)ethan-1-one **(1)** (0.005 mol) and DMF-DMF (0.7 g, 0.005 mol) in xylene (20 mL) was heated under reflux for the sufficient time of reaction (checked by TLC). After the reaction was completed, the solvent was evaporated and the residue was triturated with hexane to give a solid product that was collected by filtration and crystallized from the proper solvent to give enaminodi-sulfone **6** or enaminosulfone **12**, with isolated yields 83% and 94%, respectively.

#### 3.4.2. Method B: Microwaves Method

A mixture of 2-benzenesulfonyl-1-(4-benzenesulfonyl-phenyl)-ethanone **(4)** or 1-(4-(phenylsulfonyl)- phenyl)ethan-1-one **(1)** (0.005 mol), DMF-DMF (0.7 g, 0.005 mol), and xylene (20 mL) were mixed in a HP-500 process vial. The vail was capped properly and was irradiated by microwaves irradiation (400 W power) using pressurized conditions at 110 °C for a period of 30–40 min. the excess xylene was evaporated and the residue was triturated with hexane to give a solid product that was collected by filtration and crystallized from ethanol to give enaminosulfone **6** or enaminone **12**, with isolated yields 91% and 96%, respectively.

The physical and spectral data of the synthesized compounds **6** and **12** are listed below.

***2-benzenesulfonyl-1-(4-benzenesulfonyl-phenyl)-3-dimethylamino-propenone* (6)**, mp.: 212–214 °C, IR *ύ*: 3059 (sp^2^ C-H), 2933 (sp^3^ CH), 1624 (C=O) cm^−1^; ^1^H NMR (DMSO-*d_6_*) *δ:* 3.29 (s, 6H, 2CH_3_), 7.36–7.99 (m, 14H, Ar-H), 8.11 (s, 1H, =CH). ^13^C NMR (DMSO-*d_6_*) *δ*: 34.3 (CH_3_), 106.2, 120.6, 126.1, 127.4, 127.9, 128.5, 129.7, 130.5, 133.5, 140.5, 143.4, 143.9, 144.3, 156.1, and 187.2 (C=O). Ms *m*/*z* (%) 455 (M^+^, 39), 440 (13), 431 (100), 429 (100), 412 (25), 378 (31), 314 (13), 237 (24), 218 (35), 144 (82), 141 (26), 77 (34), and 43 (26). Anal. Calcd. For: C_23_H_21_NO_5_S_2_ (455.55) C, 60.64; H, 4.65; N, 3.07. Found: C, 60.54; H, 4.49; N, 3.12%.

***1-(4-benzenesulfonyl-phenyl)-3-dimethylamino-propenone* (12)**, mp.: 225–227 °C, IR *ύ*: 3100 (sp^2^-CH), 2921 (sp^3^-CH), 1644 (C=O) cm^−1^; ^1^H NMR (DMSO-*d_6_*) *δ:* 2.91, 3.15 (2s, 6H, 2CH_3_), 5.77 (d, *J* = 12 Hz, 1H, =CH), 7.59–7.73 and 7.89–8.11 (m, 9H, Ar-H), and 7.74 (d, *J* = 12 Hz, 1H, =CH). ^13^C NMR (DMSO-*d_6_*) *δ:* 44.6 (2CH_3_), 91.0, 127.3, 128.2, 129.7, 133.8, 140.8, 142.4, 144.7, 154.9, and 184.0 (C=O). Ms *m*/*z* (%) 315 (M+, 8), 302 (71), 272 (14), 258 (6), 245 (16), 218 (9), 140 (7), 99 (15), 77 (24), 56 (100), and 44 (88). Anal. Calcd. For: C_17_H_17_NO_3_S (315.39) C, 64.74; H, 5.43; N, 4.44. Found: C, 64.59; H, 5.21; N, 4.36%.

### 3.5. Synthesis of 2-benzenesulfonyl-1-(4-benzenesulfonyl-phenyl)-3-ethoxy-propenone **(11)**

Fusion of disulfone derivative **4** (0.4 g, 0.001 mol) and triethylorthoformate (1 mL) in round flask was achieved on hotplate for 15 min to form clear solution. After the solution was left to cool, the solid formed was collected by filtration and crystallized from ethanol to give white crystals, yield (80%), mp.: 165–167 °C, IR *ύ*: 3101 (sp^2^-CH), 2911 (sp^3^-CH), 1700 (C=O) cm^−1^.; Ms *m*/*z* (%) 456 (M^+^, 100), 274 (99), 103 (54), Anal. Calcd.For: C_23_H_20_O_6_S_2_ (456.53) C, 60.51; H, 4.42. Found: C, 60.38; H, 4.25%.

### 3.6. Synthesis of pyrazolo[1,5-a]pyrimidine derivatives **8a**–**h** and **13a**–**h**

#### 3.6.1. Thermal Methods

**Method A**: Enaminodisulfone **6** or enaminosulfone **12** (0.001 mol) was reacted with the appropriate arylazodiaminopyrazoles **7a**–**h** (0.001 mol) in 20 mL glacial acetic acid under reflux for 7 h. The reaction mixture was left to cool and the precipitated solid product was collected by filtration, washed with EtOH, dried and finally recrystallized from DMF/EtOH to afford the corresponding pyrazolo[1,5-*a*]pyrimidines **8a**–**h** or **13a**–**h**. The physical and spectral data of the synthesized compounds **8a**–**h** and **13a**–**h** are listed below.

**Method B** (for compounds **8a**–**h** only): A solution of disulfone derivative **4** (0.4 g, 0.001 mol) and an equivalent molar ratio of the appropriate arylazodiaminopyrazoles **7a**–**h** in triethylorthoformate (20 mL), was heated under reflux for 7 h. The excess solvent was removed by distillation under reduced pressure and the residue was left to cool. The precipitated solid product was collected by filtration, washed with EtOH, dried, and finally recrystallized from DMF/EtOH to afford the corresponding pyrazolo[1,5-*a*]pyrimidines **8a**–**h**.

**Method C** (for compounds **8a**–**h** only)**:** Compound **11** (0.001 mol) was reacted with the appropriate arylazodiaminopyrazoles **7a**–**h** (0.001 mol) in 20 mL glacial acetic acid under reflux for 7 h. The reaction mixture was left to cool and the precipitated solid product was collected by filtration, washed with EtOH, dried, and finally recrystallized from DMF/EtOH to afford the corresponding pyrazolo[1,5-*a*]pyrimidines **8a**–**h**.

#### 3.6.2. Microwaves Methods

**Method A**: A mixture of Enaminone **6** or enaminone **12** (0.001 mol) and the appropriate arylazodiaminopyrazoles **7a**–**h** (0.001 mol) in in 20 mL glacial acetic acid were mixed in a HP-500 process vial. The vail was capped properly and was irradiated by microwaves irradiation (800 W power) using pressurized conditions at 110 °C for 15 min. The reaction mixture was left to cool and the precipitated solid product was collected by filtration, washed with EtOH, dried, and finally recrystallized from DMF/EtOH to afford the corresponding pyrazolo[1,5-*a*]pyrimidines **8a**–**h** or **13a**–**h**. The physical and spectral data of the synthesized compounds **8a**–**h** and **13a**–**h** are listed below.

**Method B** (for compounds **8a**–**h** only): A mixture of disulfone derivative **4** (0.4 g, 0.001 mol) and an equivalent molar ratio of the appropriate arylazodiaminopyrazoles **7a**–**h** in triethylorthoformate (20 mL), was mixed in a HP-500 process vial. The vail was capped properly and was irradiated by microwaves irradiation (800 W power) using pressurized conditions at 110 °C for 15 min. The reaction mixture was left to cool and the precipitated solid product was collected by filtration, washed with EtOH, dried, and finally recrystallized from DMF/EtOH to afford the corresponding pyrazolo[1,5-*a*]pyrimidines **8a**–**h**. The physical and spectral data of the synthesized compounds **8a**–**h** and **13a**–**h** are listed below.

**Method C** (for compounds **8a**–**h** only): A mixture of compound **11** (0.001 mol) and the appropriate arylazodiaminopyrazoles **7a**–**h** (0.001 mol) in in 20 mL glacial acetic acid were mixed in a HP-500 process vial. The vail was capped properly and was irradiated by microwaves irradiation (800 W power) using pressurized conditions at 110 °C for 15 min. The reaction mixture was left to cool and the precipitated solid product was collected by filtration, washed with EtOH, dried, and finally recrystallized from DMF/EtOH to afford the corresponding pyrazolo[1,5-*a*]pyrimidines **8a**–**h**.

***6-Benzenesulfonyl-7-(4-benzenesulfonyl-phenyl)-3-(4-chloro-phenylazo)-pyrazolo[1,5-a]pyrimidin-2-ylamine* (8a)**

Yellow solid, mp.: 235–237 °C, IR *ύ*: 3464, 3362 (NH_2_), 3100 (sp^2^-CH), 2900 (sp^3^-CH), 1616 (C=N) cm^−1^; ^1^H NMR (DMSO-*d_6_*) *δ:* 7.17–7.89 (m, 12H, Ar-H and NH_2_), 7.56 (d, *J* = 8 Hz, 2H, Ar-H), 7.86 (d, *J* = 8 Hz, 2H, Ar-H), 7.92 (d, *J* = 8 Hz, 2H, Ar-H), 8.09 (d, *J* = 8 Hz, 2H, Ar-H), 9.17 (s, 1H, pyrimidine-H). ^13^C NMR (DMSO-*d_6_*) *δ:* 115.6, 120.6, 122.1, 123.0, 126.7, 127.0, 127.7, 128.9, 129.1, 129.9, 130.9, 132.3, 133.6, 134.1, 140.1, 140.5, 142.8, 144.9, 147.9, 148.7, 151.4, 153.9. Ms *m*/*z* (%) 628 (M^+^, 16), 557 (14), 521(28), 508 (93), 483 (15), 410 (38), 381 (98), 346 (16), 335 (100), 274 (17), 236 (27), 217 (13). Anal. Calcd. For: C_30_H_21_ClN_6_O_4_S_2_ (629.11) C, 57.27; H, 3.36; N, 13.36. Found: C, 57.09; H, 3.16; N, 13.27%.

***6-Benzenesulfonyl-7-(4-benzenesulfonyl-phenyl)-3-(3-methyl-phenylazo)-pyrazolo[1,5-a]pyrimidin-2-ylamine* (8b)**

Pale brown solid, mp.: 233–235 °C, IR *ύ*: 3459, 3337 (NH_2_), 3096 (sp^2^-CH), 2980 (sp^3^-CH), 1609 (C=N) cm^−1^; ^1^H NMR (DMSO-*d_6_*) *δ:* 2.39 (s, 3H, CH_3_), 7.15–7.80 (m, 16H, Ar-H and NH_2_), 7.99 (d, *J* = 9 *Hz,* 2H, Ar-H), 8.09 (d, *J* = 9 Hz, 2H, Ar-H), and 9.16 (s, 1H, pyrimidine-H).^13^C NMR (DMSO-*d_6_*) *δ:* 20.9 (CH_3_), 115.4, 119.2, 121.5, 121.8, 126.8, 127.0, 127.8, 128.9, 129.1, 130.0, 130.2, 131.0, 132.4, 133.7, 134.2, 138.5, 140.2, 140.6, 142.7, 144.9, 147.8, 148.5, 152.7, and 154.0. Ms *m*/*z* (%) 610 (M^+^ + 2, 13), 608 (M^+^, 23), 593 (14), 541 (100), 532 (23), 517 (32), 489 (16), 391 (34), 326 (9), 235 (16), 217 (9), 140 (15), 129 (90), 95 (43), and 76 (10). Anal. Calcd. For: C_31_H_24_N_6_O_4_S_2_ (608.69) C, 61.17; H, 3.97; N, 13.81. Found: C, 61.03; H, 3.82; N, 13.69%.

***6-Benzenesulfonyl-7-(4-benzenesulfonyl-phenyl)-3-(3-chloro-phenylazo)-pyrazolo[1,5-a]pyrimidin-2-ylamine* (8c)**

Yellow crystals, mp.: 260–262 °C, IR *ύ*: 3408, 3280 (NH_2_), 3068 (sp^2^-CH) 1616 (C=N); ^1^H NMR (DMSO-*d_6_*) *δ:* 3.57 (s, 2H, NH_2_), 7.16 (t, *J* = 7.65 Hz, 2H, Ar-H), 7.30 (d, *J* = 8.5 Hz, 2H, Ar-H), 7.46 (d, J = 7.65 Hz, 2H, Ar-H), 7.53–7.86 (m, 7H, Ar-H), 7.95 (s, 1H, Ar-H), 7.99 (d, *J* = 7.65 Hz, 2H, Ar-H), 8.10 (d, *J* = 7.65 Hz, 2H, Ar-H), and 9.19 (s, 1H, N=CH). ^13^C NMR (DMSO-*d_6_*) δ: 115.8, 120.4, 120.9, 122.4, 126.9, 127.1, 127.9, 128.8, 129.0, 130.0, 130.1, 131.0, 132.4, 133.8, 134.0, 134.3, 140.1, 140.6, 142.8, 145.1, 148.1, 148.8, 153.9, and 154.0. Ms *m*/*z* (%) 628 (M^+^−1, 18), 552 (27), 520 (11), 483 (17), 409 (26), 345 (15), 274 (10), 236 (15), 216 (17), 111 (8), and 72 (100). Anal. Calcd. For: C_30_H_21_ClN_6_O_4_S_2_ (629.11) C, 57.27; H, 3.36; N, 13.36. Found: C, 57.08; H, 3.21 N, 13.29%.

***6-Benzenesulfonyl-7-(4-benzenesulfonyl-phenyl)-3-(2-chloro-phenylazo)-pyrazolo[1,5-a]pyrimidin-2-ylamine* (8d)**

Yellow solid, mp.: 250–252 °C, IR *ύ*:br. 3458 (NH_2_), 1612 (C=N); ^1^H NMR (DMSO-*d_6_*) 5.40 (s, 2H, NH_2_), 7.17–7.82 (m, 14H, Ar-H), 7.98 (d, *J* = 8 Hz, 2H, Ar-H), 8.09 (d, *J* = 8 Hz, 2H, Ar-H), and 9.22 (s, 1H, pyrimidine-H). ^13^C NMR (DMSO-*d_6_*) *δ:* 111.9, 116.4, 116.7, 122.7, 126.8, 127.8, 127.1, 127.6, 128.1, 128.9, 130.3, 130.7, 132.2, 132.3, 134.2, 138.0, 139.9, 140.5, 142.8, 145.2, 147.8, 149.0, 153.7, and 156.7. Ms *m*/*z* (%) 629 (M^+^, 32), 579 (100), 552 (49), 519 (17), 504 (35), 489 (15), 412 (7), 346 (34), 275 (8), 141 (6), and 111 (19). Anal. Calcd. For: C_30_H_21_ClN_6_O_4_S_2_ (629.11) C, 57.27; H, 3.36; N, 13.36. Found: C, 57.07; H, 3.30; N, 13.15%.

***6-Benzenesulfonyl-7-(4-benzenesulfonyl-phenyl)-3-(3-methoxy-phenylazo)-pyrazolo[1,5-a]pyrimidin-2-ylamine* (8e)**

Dark yellow solid, mp.: 200–202 °C, IR *ύ* br. 3450 (NH_2_), 1618 (C=N); ^1^H NMR (DMSO-*d_6_*) *δ:* 3.57 (s, 3H, OCH_3_), 5.40 (s, 2H, NH_2_), 7.56–8.12 (m, 18H, Ar-H), and 9.17 (s, 1H, pyrimidine-H). Ms *m*/*z* (%) 624 (M^+^, 50), 453 (87), 439 (99), 318 (94), and 274 (100). Anal. Calcd.For: C_31_H_24_N_6_O_5_S_2_ (624.69) C, 59.60; H, 3.87; N, 13.45. Found: C, 59.46; H, 3.73; N, 13.21%.

***6-Benzenesulfonyl-7-(4-benzenesulfonyl-phenyl)-3-phenylazo-pyrazolo[1,5-a]pyrimidin-2-ylamine* (8f)**

Dark yellow solid, mp.: 270–272 °C, IR *ύ*: br, 3456 (NH_2_), 1614 (C=N); ^1^H NMR (DMSO-*d_6_*) *δ*: 7.15–7.89 (m, 15H, Ar–H), 7.98 (d, *J* = 8 Hz, 2H, Ar-H), 8.09 (d, *J* = 8 Hz, 2H, Ar-H), and 9.18 (s, 1H, pyrimidine-H). ^13^C NMR (DMSO-*d_6_*) *δ:* 115.4, 121.6, 121.9, 126.9, 127.1, 127.9, 129.0, 129.2, 129.6, 130.1, 131.0, 132.5, 133.8, 134.3, 140.2, 140.6, 142.8, 145.0, 147.9, 148.6, 152.7, and 154.0. Ms *m*/*z* (%) 594 (M^+^, 20), 580 (18), 523 (22), 490 (32), 457 (49), 375 (100), 273 (28), 218 (28), 142 (24), and 77 (27). Anal. Calcd. For: C_30_H_22_N_6_O_4_S_2_ (594.66) C, 60.59; H, 3.73; N, 14.13. Found: C, 60.46; H, 3.61; N, 14.02%.

***6-Benzenesulfonyl-7-(4-benzenesulfonyl-phenyl)-3-(2-nitro-phenylazo)-pyrazolo[1,5-a]pyrimidin-2-ylamine* (8g)**

Dark red solid, mp.: 285–287 °C, IR *ύ*: 3446, 3340 (NH_2_), 1620, 1585 (C=N).; ^1^H NMR (DMSO-*d_6_*) *δ:* 7.16–8.12 (m, 20H, Ar–H), 9.23 (s, 1H, pyrimidine-H). ^13^C NMR (DMSO-*d_6_*) *δ:*117.3, 117.4, 120.5, 123.3, 124.4, 126.8, 127.1, 127.8, 128.9, 129.5, 129.9, 130.9, 132.2, 133.6, 133.8, 134.2, 139.9, 140.6, 142.9, 144.6, 145.3, 146.0, 149.4, and 153.6. Ms *m*/*z* (%) 639 (M^+^, 23), 620 (19), 563 (15), 516 (13), 494 (14), 438 (99), 423 (32), 148 (21), 137 (17), 123 (12), 77 (8), and 60 (100). Anal. Calcd. For: C_30_H_21_N_7_O_6_S_2_ (639.66) C, 56.33; H, 3.31; N, 15.33. Found: C, 56.18; H, 3.29; N, 15.08%.

***6-Benzenesulfonyl-7-(4-benzenesulfonyl-phenyl)-3-(4-methoxy-phenylazo)-pyrazolo[1,5-a]pyrimidin-2-ylamine* (8h)**

Pale brown solid, mp.: 220–222 °C (EtOH), IR *ύ*: br. 3450 (NH_2_), 1616 (C=N); ^1^H NMR (DMSO-*d_6_*) *δ:* 3.83 (s, 3H, OCH_3_), 7.05 (d, *J* = 8 Hz, 2H, Ar-H), 7.14–7.47 (m, 10H, Ar-H), 7.53 (s, 2H, NH_2_), 7.85 (d, *J* = 8 Hz, 2H, Ar-H), 7.97 (d, *J* = 8 Hz, 2H, Ar-H), 8.09 (d, *J* = 8 Hz, 2H, Ar-H), and 9.13 (s, 1H, pyrimidine-H). ^13^C NMR (DMSO-*d_6_*) *δ:* 55.5, 114.4, 115.0, 121.4, 123.1, 126.8, 127.0, 127.8, 128.9, 129.9, 130.9, 132.5, 133.6, 134.1, 140.3, 140.6, 142.8, 144.8, 146.8, 147.2, 148.2, 154.2, and 160.6. Ms *m*/*z* (%) 624 (M^+^, 24), 550 (16), 488 (18), 407 (47), 273 (38), 217 (44), 159 (100), 133 (6), and 80 (28). Anal. Calcd. For: C_31_H_24_N_6_O_5_S_2_ (624.69) C, 59.60; H, 3.87; N, 13.45. Found: C, 59.42; H, 3.70; N, 13.31%.

***7-(4-Benzenesulfonyl-phenyl)-3-(4-chloro-phenylazo)-pyrazolo[1,5-a]pyrimidin-2-ylamine* (13a)**

Orange crystals, mp.: 280–282 °C (EtOH), IR *ύ*: 3421, 3298 (NH_2_), 3099 (sp^2^-CH), 2900 (sp^3^-CH), 1616 (C=N) cm^−1^; ^1^H NMR (DMSO-*d_6_*) *δ*: 7.30 (d, *J* = 3.9 Hz, 1H, Pyrimidine-H), 7.52 (d, *J* = 9 Hz, 2H, Ar-H), 7.68–7.70 (m, 7H, Ar-H and NH_2_), 7.83 (d, *J* = 9 Hz, 2H, Ar-H), 8.04–8.25 (m, 4H, Ar-H), 8.63 (d, *J* = 3.9 Hz, and 1H, Pyrimidine-H). ^13^C NMR (DMSO-*d_6_*) *δ:* 110.0, 114.9, 122.8, 127.4, 127.7, 129.18, 130.0, 131.0, 132.7, 134.2, 135.2, 140.6, 142.9, 143.5, 147.6, 150.9, 151.7, and 151.9. Ms *m*/*z* (%) 488 (M^+^, 14), 414 (14), 363 (24), 270 (17), 255 (29), 218 (13), 140 (9), 131 (100), 121 (74), 110 (24), and 102 (29). Anal. Calcd. For: C_24_H_17_ClN_6_O_2_S (488.95) C, 58.95; H, 3.50; N, 17.19. Found: C, 58.76; H, 3.41; N, 17.06%.

***7-(4-Benzenesulfonyl-phenyl)-3-m-tolylazo-pyrazolo[1,5-a]pyrimidin-2-ylamine* (13b)**

Red solid, mp.: 235–237 °C (AcOH), IR *ύ*: 3421, 3274 (NH_2_), 3165 (sp^2^-CH), 2919 (sp^3^-CH), 1616 (C=N) cm^−1^; ^1^H NMR (DMSO-*d_6_*) *δ:* 2.39 (s, 3H, CH_3_), 7.18 (d, *J* = 7.65 Hz, 2H, Ar-H), 7.25 (s, 2H, NH_2_), 7.27 (d, *J* = 5.1 Hz, 1H, Pyrimidine-H), 7.37 (t, *J* = 7.65 Hz, 2H, Ar-H), 7.62 (d, *J* = 7.65 Hz, 1H, Ar-H), 7.64 (s, 1H, Ar-H), 7.69 (t, *J* = 7.65 Hz, 1H, Ar-H), 7.75 (t, *J* = 7.65 Hz, 1H, Ar-H), 8.06 (d, *J* = 7.65 Hz, 1H, Ar-H), 8.18 (d, *J* = 7.65 Hz, 2H, Ar-H), 8.23 (d, *J* = 7.65 Hz, 2H, Ar-H), 8.63 (d, *J* = 5.1 Hz, 1H, Pyrimidine-H). ^13^C NMR (DMSO-*d_6_*) *δ:* 20.9 (CH_3_), 109.5, 114.6, 118.9, 120.5, 121.2, 127.3, 127.6, 128.8, 129.3, 129.9, 134.0, 135.2, 138.4, 140.6, 142.9, 143.3, 147.5, 150.6, 151.8, 152.9. Ms *m*/*z* (%) 468 (M^+^, 8), 454 (14), 395 (11), 377 (15), 311 (12), 250 (7), 215 (7), 171 (74), 142 (2), 91 (14), 81 (100). Anal. Calcd. For: C_25_H_20_N_6_O_2_S (468.53) C, 64.09; H, 4.30; N, 17.94. Found: C, 63.89; H, 4.21; N, 17.85%.

***7-(4-Benzenesulfonyl-phenyl)-3-(3-chloro-phenylazo)-pyrazolo[1,5-a]pyrimidin-2-ylamine* (13c)**

Red crystals, mp.: 245–247 °C (AcOH), IR *ύ*: 3421, 3274 (NH_2_), 3067 (sp^2^-CH), 1616 (C=N) cm^−1^; ^1^H NMR (DMSO-*d_6_*) *δ:* 7.26–8.23 (m, 16H, Ar-H and NH_2_), 8.61 (d, *J* = 4.5 Hz, 1H, pyrimidine-H).^13^C NMR (DMSO-*d_6_*) *δ*: 110.0, 115.1, 120.2, 120.3, 127.3, 127.6, 127.8, 129.9, 130.5, 130.9, 133.8, 134.0, 135.1, 140.6, 142.9, 143.4, 147.7, 150.9, 151.9, and 154.2. Ms *m*/*z* (%) 488 (M^+^, 8), 458 (19), 412 (19), 377 (11), 273 (44), 249 (100), 218(7), 161 (14), 143 (37), 111 (59), and 108 (74). Anal. Calcd. For: C_24_H_17_ClN_6_O_2_S (488.95) C, 58.95; H, 3.50; N, 17.19. Found: C, 58.88; H, 3.41; N, 17.01%.

***7-(4-Benzenesulfonyl-phenyl)-3-(2-chloro-phenylazo)-pyrazolo[1,5-a]pyrimidin-2-ylamine* (13d)**

Orange crystals, mp.: 280–282 °C (AcOH), IR *ύ*: 3398, 3298 (NH_2_), 3164 (sp^2^-CH), 2997 (sp^3^-CH), 1615 (C=N) cm^−1^; ^1^H NMR (DMSO-*d_6_*) *δ:* 7.32 (d, *J* = 4 Hz, 1H, pyrimidine-H), 7.46 (s, 2H, NH_2_), 7.67–8.24 (m, 13H, Ar-H), and 8.66 (d, *J* = 4 Hz, 1H, pyrimidine-H). ^13^C NMR (DMSO-*d_6_*) *δ:* 110.6, 116.4, 116.43, 127.4, 127.7, 128.0, 129.8, 130.0, 130.3, 131.1, 131.5, 134.2, 135.1, 140.6, 143.0, 143.8, 147.8, 148.2, 151.3, and 151.9. Ms *m*/*z* (%) 488 (M^+^, 8), 452 (13), 411 (100), 378 (28), 364 (20), 272 (30), 254 (17), 213 (25), 111 (26), and 90 (27). Anal. Calcd. For: C_24_H_17_ClN_6_O_2_S (488.95) C, 58.95; H, 3.50; N, 17.19. Found: C, 58.82; H, 3.34; N, 17.06%.

***7-(4-Benzenesulfonyl-phenyl)-3-(3-methoxy-phenylazo)-pyrazolo[1,5-a]pyrimidin-2-ylamine* (13e)**

Red solid, mp.: 258–260 °C (AcOH), IR *ύ*: 3437, 3298 (NH_2_), 3155 (sp^2^-CH), 2909 (sp^3^-CH), 1612 (C=N) cm^−1^; ^1^H NMR (DMSO-*d_6_*) *δ:* 3.83 (s, 3H, OCH_3_), 6.94–6.95 (m, 1H, Ar-H), 7.27 (s, 2H, NH_2_), 7.29 (d, *J* = 4.5 Hz, 1H, pyrimidine-H), 7.39–7.44 (m, 3H, Ar-H), 7.68 (t, *J* = 7.65 Hz, 2H, Ar-H), 7.74 (t, *J* = 6.8 Hz, 1H, Ar-H), 8.06 (d, *J* = 7.65 Hz, 2H, Ar-H), 8.18 (d, *J* = 8.5 Hz, 2H, Ar-H), 8.23 (d, *J* = 8.5 Hz, 2H, Ar-H), and 8.64 (d, *J* = 4.5 Hz, 1H, pyrimidine-H). ^13^C NMR (DMSO-*d_6_*) *δ:* 55.2, 105.5, 109.6, 114.1, 114.6, 114.9, 127.3, 127.6, 129.7, 129.9, 130.9, 134.0, 135.2, 140.6, 142.9, 143.3, 147.5, 150.7, 151.9, 154.2, and 160.0. Ms *m*/*z* (%) 484 (M^+^, 15), 454 (24), 407 (11), 379 (17), 360 (100), 349 (12), 327 (10), 267 (7), 218 (15), 160 (7), 136 (41), and 78(17). Anal. Calcd. For: C_25_H_20_N_6_O_3_S (484.53) C, 61.97; H, 4.16; N, 17.34. Found: C, 61.76; H, 4.00; N, 17.15%.

***7-(4-Benzenesulfonyl-phenyl)-3-phenylazo-pyrazolo[1,5-a]pyrimidin-2-ylamine* (13f)**

Red solid, mp.: 215–217 °C (AcOH), IR *ύ*: 3420, 3270 (NH_2_), 3162 (sp^2^-CH), 2998 (sp^3^-CH), 1616 (C=N) cm^−1^; ^1^H NMR (DMSO-*d_6_*) *δ:* 7.22–8.24 (m, 17H, Ar-H and NH_2_), 8.60 (d, *J* = 4.5 Hz, 1H, pyrimidine-H). ^13^C NMR (DMSO-*d_6_*) *δ:* 114.7, 121.1, 127.3, 127. 6, 129.0, 129.2, 129.9, 130.9, 131.1, 134.0, 135.1, 140.6, 142.9, 143.3, 147.5, 150.6, 151.8, and 152.9. Ms *m*/*z* (%) 454 (M^+^, 19), 438 (27), 364 (7), 361 (27), 349 (16), 313 (14), 237 (10), 217 (14), 208 (27), and 106 (100). Anal. Calcd. For: C_24_H_18_N_6_O_2_S (454.50) C, 63.42; H, 3.99; N, 18.49. Found: C, 63.32; H, 3.88; N, 18.32%.

***7-(4-Benzenesulfonyl-phenyl)-3-(2-nitro-phenylazo)-pyrazolo[1,5-a]pyrimidin-2-ylamineylamine* (13g)**

Red solid, mp.: 265–267 °C (AcOH), IR *ύ*: 3410, 3294 (NH_2_), 3165 (sp^2^-CH), 2900 (sp^3^-CH), 1617 (C=N) cm^−1^; ^1^H NMR (DMSO-*d_6_*) *δ:* 7.46 (d, *J* = 4.5 Hz, 1H, pyrimidine-H), 7.60 (t, *J* = 7 Hz, 1H, Ar-H), 7.73–7.83 (m, 6H, Ar-H and NH_2_), 7.95 (dd, *J* = 8, 1.7Hz, 1H, Ar-H), 8.05 (dd, *J* = 8, 1.7Hz, 1H, Ar-H), 8.11 (dd, *J* = 8, 1.7Hz, 2H, Ar-H), 8.24 (d, *J* = 8.5 Hz, 2H, Ar-H), 8.27 (d, *J* = 8.5 Hz, 2H, Ar-H), and 8.76 (d, *J* = 4.5 Hz, 1H, pyrimidine-H). Ms *m*/*z* (%) 499 (M^+^, 58), 453 (11), 422 (28), 378 (27), 362 (5), 218 (6), 208 (100), 151(11), 142 (5), 122 (10), and 77 (30). Anal. Calcd. For: C_24_H_17_N_7_O_4_S (499.50) C, 57.71; H, 3.43; N, 19.63. Found: C, 57.56; H, 3.40; N, 19.52%.

***7-(4-Benzenesulfonyl-phenyl)-3-(4-methoxy-phenylazo)-pyrazolo[1,5-a]pyrimidin-2-ylamine* (13h)**

Red, yield (94%), mp.: 255–257 °C (AcOH), IR *ύ*: 3447, 3337 (NH_2_), 3066 (sp^2^-CH), 2900 (sp^3^-CH), 1611 (C=N) cm^−1^; ^1^H NMR (DMSO-*d_6_*) *δ:* 3.83 (s, 3H, OCH_3_), 7.06 (d, *J* = 8.5 Hz, 2H, Ar-H), 7.17 (s, 2H, NH_2_), 7.24 (d, *J* = 5.1 Hz, 1H, pyrimidine-H), 7.68 (t, *J* = 7.65 Hz, 2H, Ar-H), 7.74 (t, *J* = 7.65 Hz, 1H, Ar-H), 7.81 (d, *J* = 8.5 Hz, 2H, Ar-H), 8.06 (d, *J* = 8.5 Hz, 2H, Ar-H), 8.18 (d, *J* = 8.5 Hz, 2H, Ar-H), 8.23 (d, *J* = 8.5 Hz, 2H, Ar-H), and 8.61(d, *J* = 4.5 Hz, 1H, pyrimidine-H). ^13^C NMR (DMSO-*d_6_*) *δ:* 55.4, 109.1, 114.1, 114.3, 122.6, 127.3, 127.6, 129.9, 130.8, 134.0, 135.3, 140.6, 142.9, 143.2, 147.1, 147.2, 150.4, 151.9, 159.9. Ms *m*/*z* (%) 484 (M^+^, 22), 454 (16), 406 (23), 377 (24), 351 (90), 327 (22), 251 (50), 134(5), 124 (43), 105 (19), and 58 (100). Anal. Calcd. For: C_25_H_20_N_6_O_3_S (484.53) C, 61.97; H, 4.16; N, 17.34. Found: C, 61.78; H, 4.03; N, 17.25%.

### 3.7. Biological Methods

#### Antimicrobial Activity Test

The antimicrobial activity of the synthesized compounds have been determined using the agar diffusion well method which is a suitable for such biological activity measurement. Culture collection of the Regional Center for Mycology and Biotechnology (RCMB), Al-Azhar University, Cairo, Egypt provided all strains in this study. For fungi, the microbes’ inoculums were spread using sterile cotton swab with uniform manner on a sterile petri dish malt extract agar. In case of bacteria, the microbes’ inoculums were spread on the nutrient agar. 100 μL of a given sample was added to each well which is ten mm diameter holes cut in the agar gel, twenty mm apart from each other). All systems prepared were incubated for 1–2 days at 37 °C for antibacterial activity measurements and at a temperature of 28 °C for antifungal measurements. The microorganism’s growth was observed after the latter incubation. The inhibition zone of the bacterial and fungal growth have been measured as IZD in millimeter. Finally, all the mentioned tests were performed in triplicate for all compounds.

In case of estimation the MIC of the examined samples, micro-dilution test was performed in 96-well plates. Two-fold dilutions of each sample were prepared in the test wells, the final drug concentrations being (125–0.004) µg/mL, control wells were prepared with culture medium only and microbial suspension only. The plates were sealed and incubated for 24 h at 37 °C for bacteria and for 48 h at 28 °C for fungi, after each incubation time, MIC was detected as the lowest sample concentration that prevented microbial growth. Each MIC was determined three times.

## 4. Conclusions

In conclusion, we have synthesized new series of pyrazolo[1,5-*a*]pyrimidine derivatives incorporated phenylsulfonyl and arylazo moieties using a simple methodology. The synthetic methodology included the use of conventional heating and microwaves irradiation under pressurized conditions in a safe manner. The antimicrobial activities of novel compounds are evaluated and three compounds **13c**, **13d**, and **13g** demonstrated the highest antibacterial activity against all Gram-positive and -negative bacteria. Other sulfone derivatives showed fair to low antibacterial and antifungal activities. In general, derivatives containing one phenylsulfonyl group are more effective against all most antibacterial and antifungal species used than that contain two phenylsulfonyl groups.

## Figures and Tables

**Table 1:** A comparison between the conventional and microwave heating for the synthesis of compounds **8a**–**h**.

Compound No.	Ar	Conventional Heating	Microwave Heating
		Yield%	Yield%
**8a**	4-ClC_6_H_4_-	77	95
**8b**	3-CH_3_C_6_H_4_-	85	95
**8c**	3-ClC_6_H_4_-	77	91
**8d**	2-ClC_6_H_4_-	78	90
**8e**	3-CH_3_OC_6_H_4_-	80	90
**8f**	C_6_H_5_-	75	90
**8g**	2-NO_2_C_6_H_4_-	90	91
**8h**	4-CH_3_OC_6_H_4_-	95	95

**Table 2:** A comparison between the conventional and microwave heating for the synthesis of compounds **13a**–**h**.

Compound No.	Ar	Conventional Heating	Microwave Heating
		Yield%	Yield%
**13a**	4-ClC_6_H_4_-	92	98
**13b**	3-CH_3_C_6_H_4_-	90	97
**13c**	3-ClC_6_H_4_-	90	94
**13d**	2-ClC_6_H_4_-	85	97
**13e**	3-CH_3_O C_6_H_4_-	90	93
**13f**	C_6_H_5_-	80	90
**13g**	2-NO_2_C_6_H_4_-	92	96
**13h**	4-CH_3_O C_6_H_4_-	94	98

**Table 3:** The antifungal activity of the tested derivatives **8a**–**d**, **8h**, **13a**, and **13c**–**h**.

Compound No.	Aspergillus Niger *	Geotrichum Candidum *
**8a**	15.5 ± 1.2	17.4 ± 0.72
**8b**	15.2 ± 0.60	17.2 ± 0.63
**8c**	17.6 ± 1.2	18.5 ± 0.63
**8d**	18.3 ± 0.63	19.3 ± 0.58
**8h**	22.4 ± 2.1	24.3 ± 2.1
**13a**	23.1 ± 0.72	22.6 ± 0.72
**13c**	25.1 ± 1.2	22.6 ± 1.2
**13d**	21.4 ± 1.2	25.2 ± 1.2
**13e**	22.3 ± 1.2	20.4 ± 0.58
**13f**	22.6 ± 0.72	23.6 ± 1.20
**13g**	26.3 ± 0.63	23.2 ± 0.63
**13h**	19.2 ± 0.72	17.3 ± 0.72
***Amphotericin B***	23.3 ± 0.58	25.2 ± 0.72

* The zone of inhibition (IZD) is measured in millimeter.

**Table 4:** The antimicrobial activity of the tested derivatives (μg/mL) against Gram-positive bacteria.

Compound No.	*S. aureus*	*S. epidermidis*	*B. subtilis*	*S. pyogenes*
**8a**	16.3 ± 0.63	15.8 ± 0.58	16.9 ± 0.53	NA
**8b**	18.4 ± 0.85	15.7 ± 1.2	18.6 ± 0.63	NA
**8c**	17.6 ± 0.63	16.4 ± 0.72	21.3 ± 0.53	NA
**8d**	19.7 ± 0.58	18.3 ± 0.58	20.7 ± 1.2	NA
**8h**	21.1 ± 1.2	20.8 ± 0.67	24.3 ± 0.58	NA
**13a**	20.8 ± 0.43	20.6 ± 0.58	25.4 ± 0.53	NA
**13c**	23.4 ± 0.63	21.8 ± 0.72	23.6 ± 0.63	NA
**13d**	22.7 ± 0.63	22.6 ± 0.72	25.5 ± 0.63	NA
**13e**	19.8 ± 0.63	16.7 ± 0.58	23.6 ± 0.53	NA
**13f**	21.4 ± 0.58	17.7 ± 0.72	26.5 ± 0.58	NA
**13g**	22.7 ± 1.2	22.3 ± 0.58	23.7 ± 0.72	NA
**13h**	13.5 ± 1.2	15.3 ± 0.44	18.2 ± 0.58	NA
***Ampicillin***	23.7 ± 0.63	22.4 ± 1.2	32.4 ± 0.72	24.5 ± 0.63

* The zone of inhibition (IZD) is measured in millimeter.

**Table 5:** The antimicrobial activity of the tested derivatives (μg/mL) against Gram-negative bacteria.

Compound No.	*P. aeruginosa*	*E. coli*	*K. pneumoniae*	*S. typhimurium*
**8a**	NA	15.6 ± 1.2	11.8 ± 0.44	18.5 ± 0.72
**8b**	NA	17.5 ± 0.58	14.8 ± 1.2	16.7 ± 0.63
**8c**	NA	18.7± 1.2	15.9 ± 1.2	17.4 ± 0.58
**8d**	NA	18.6 ± 0.63	17.6 ± 0.58	20.2 ± 0.72
**8h**	NA	23.2 ± 0.58	21.3 ± 0.58	19.8 ± 1.2
**13a**	NA	22.4 ± 0.53	20.4 ± 0.53	21.6 ± 0.63
**13c**	NA	24.3 ± 1.2	22.5 ± 1.2	26.3 ± 0.58
**13d**	NA	25.7 ± 1.2	26.6 ± 1.2	26.2 ± 0.58
**13e**	NA	19.8 ± 1.2	18.4 ± 0.53	21.1 ± 0.63
**13f**	NA	23.2 ± 0.72	19.5 ± 0.63	22.5 ± 0.63
**13g**	NA	25.5 ± 1.2	23.3 ± 1.2	26.6 ± 0.72
**13h**	NA	19.3 ± 0.63	16.3 ± 0.63	19.3 ± 0.58
**Gentamicin**	22.3 ± 0.58	25.4 ± 1.2	2.6 ± 0.63	23.3 ± 0.58

* The zone of inhibition (IZD) is measured in millimeter.

**Table 6:** Minimum inhibitory concentration (μg/mL) for compounds **15c**, **15d**, and **15g**.

Compound No.	13c	13d	13g	Reference
**Fungi**				***Amphotericin B***
***Aspergillus niger***	3.9	0.98	1.95	0.98
**Geotrichum candidum**	7.81	1.95	3.9	0.49
***G^+^ Bacteria***				***Ampicillin***
***St. aureus***	3.9	3.9	15.63	0.98
***St. epidermidis***	15.63	7.81	31.25	1.95
***B. subtilis***	0.98	0.49	1.95	0.49
***St. pyogenes***	NA	NA	NA	0.49
***G^-^ Bacteria***				***Gentamicin***
***P. aeruginosa***	NA	NA	NA	1.95
***E. coli***	3.9	0.49	3.9	0.49
***K. pneumoniae***	7.81	3.9	15.63	0.98
***S. typhimurium***	3.9	1.95	3.9	0.98
